# Self-Consistent Hybrid Functional Calculations: Implications for Structural, Electronic, and Optical Properties of Oxide Semiconductors

**DOI:** 10.1186/s11671-016-1779-9

**Published:** 2017-01-06

**Authors:** Daniel Fritsch, Benjamin J. Morgan, Aron Walsh

**Affiliations:** Department of Chemistry, University of Bath, Claverton Down, BA2 7AY, Bath, UK

**Keywords:** Density functional theory, Hybrid functionals, Semiconducting oxides, Dielectric functions, 71.15.Mb, 71.20.Nr, 78.20.Bh

## Abstract

The development of new exchange-correlation functionals within density functional theory means that increasingly accurate information is accessible at moderate computational cost. Recently, a newly developed self-consistent hybrid functional has been proposed (Skone et al., Phys. Rev. B 89:195112, 2014), which allows for a reliable and accurate calculation of material properties using a fully ab initio procedure. Here, we apply this new functional to wurtzite ZnO, rutile SnO_2_, and rocksalt MgO. We present calculated structural, electronic, and optical properties, which we compare to results obtained with the PBE and PBE0 functionals. For all semiconductors considered here, the self-consistent hybrid approach gives improved agreement with experimental structural data relative to the PBE0 hybrid functional for a moderate increase in computational cost, while avoiding the empiricism common to conventional hybrid functionals. The electronic properties are improved for ZnO and MgO, whereas for SnO_2_ the PBE0 hybrid functional gives the best agreement with experimental data.

## Background

Metal oxides exhibit many unique structural, electronic, and magnetic properties, making them useful for a broad range of technological applications. Metal oxides are exclusively used as transparent conducting oxides (TCOs) [1], find applications as building blocks in artificial multiferroic heterostructures [2] and as spin-filter devices [3], and even include a huge class of superconducting materials. To develop new materials for specific applications, it is necessary to have a detailed understanding of the interplay between the chemical composition of different materials, their structure, and their electronic, optical, or magnetic properties.

For the development of new functional oxides, computational methods that allow theoretical predictions of structural and electronic properties have become an increasingly useful tool. When optical or electronic properties are under consideration, electronic structure methods are necessary, with the most popular approach for solids being density functional theory (DFT). DFT has proven hugely successful in the calculation of structural properties of condensed matter systems and the electronic properties of simple metals [4]. The earliest developed approximate exchange-correlation functionals, however, face limitations, for example severely underestimating band gaps of semiconductors and insulators.

Over the last decade, several new, more accurate, exchange-correlation functionals have been proposed. Increased predictive accuracy often comes with an increased computational cost, and the adoption of these more accurate functionals has only been made possible through the continued increase in available computational power. One such more accurate, and more costly, approach is to use so-called *hybrid* functionals. These are constructed by mixing a fraction of Hartree-Fock exact-exchange with the exchange and correlation terms from some underlying DFT functional. Calculated material properties, such as lattice parameters and band gaps, however depend on the precise proportion of Hartree-Fock exact-exchange, *α*. Typical hybrid functionals treat *α* as a fixed *empirical* parameter, chosen by intuition and experimental calibration. A recently proposed *self-consistent* hybrid functional approach for condensed systems [5] avoids this empiricism and allows parameter-free hybrid functional calculations to be performed. In this approach, the amount of Hartree-Fock exact-exchange is identified as the inverse of the dielectric constant, with this constraint achieved by performing an iterative sequence of calculations to self-consistency.

Here we apply this new self-consistent hybrid functional to wurtzite ZnO and rutile SnO_2_, both materials with potential applications as TCOs, and MgO, a wide band gap insulator [6]. We examine the implications of the self-consistent hybrid functional for the structural, electronic, and optical properties. In the next section, we present the theoretical background, describe the self-consistent hybrid functional, and give the computational details. We then present results for the structural, electronic, and optical properties for ZnO, SnO_2_, and MgO, and compare these to data calculated using alternative exchange-correlation functionals and from experiments. The paper concludes with a summary and an outlook.

## Methods

### Density functional theory and hybrid functionals

DFT is a popular and reliable tool to theoretically describe the electronic structure of both crystalline and molecular systems. DFT provides a mean-field simplification of the many-body Schrödinger equation. The central variable is the electron density $n(\vec {r}) = \psi ^{*}(\vec {r})\psi (\vec {r})$, determined from the electronic wavefunctions $\psi (\vec {r})$, and the Hamiltonian is described as a functional of the $n(\vec {r})$. Within the generalised Kohn-Sham scheme, the potential is

1 $$ v_{\text{GKS}}(\vec{r}, \vec{r}^{\prime}) = v_{\mathrm{H}}(\vec{r}) + v_{\text{xc}}(\vec{r},\vec{r}^{\prime}) + v_{\text{ext}}(\vec{r}).  $$

The Hartree potential, *v*_H_(**r**), and the external potential, *v*_ext_(**r**), are in principle known. The exchange-correlation potential, $v_{\text {xc}}(\vec {r},\vec {r}^{\prime })$, however, is not and must be approximated. Most successful early approximations make use of the local density approximation and the semilocal generalised gradient approximation (GGA), for example, in the parametrisation of Perdew, Burke, and Ernzerhof (PBE) [7]. These approximations already allowed reliable descriptions of structural properties within the computational resources available at the time, but lacked accuracy when determining band energies, especially fundamental band gaps, and *d* valence band widths of semiconductors. These properties are particularly important for reliable calculations of electronic and optical behaviours of semiconductors.

In recent years, so-called *hybrid* functionals have gained in popularity. In a hybrid functional, some proportion of the local exchange-correlation potential is replaced by Hartree-Fock exact-exchange terms, giving a better description of electronic properties. The explicit inclusion of exact-exchange Hartree-Fock terms make these calculations computationally much more demanding compared to the earlier GGA calculations, and hybrid functional calculations have become routine only in recent years. The fraction of Hartree-Fock exact-exchange admixed in these hybrid functionals, *α*, is usually justified on experimental or theoretical grounds, and then fixed for a specific functional. This adds an empirical parameter and forfeits the ab initio nature of the calculations. One popular choice of *α*=0.25 is realised in the PBE0 functional [8].

In this work, we are concerned with full-range hybrid functionals, for which the generalised nonlocal exchange-correlation potential is

2 $$ v_{\text{xc}}(\vec{r},\vec{r}^{\prime}) = \alpha v_{\mathrm{x}}^{\text{ex}}(\vec{r},\vec{r}^{\prime}) + (1-\alpha) v_{\mathrm{x}}(\vec{r}) + v_{\mathrm{c}}(\vec{r}).  $$

A common approach is to select *α* to reproduce the experimental band gap of solid state systems. Apart from adding an empirical parameter into the calculations, fitting the band gap of a material requires reliable experimental data. Moreover, this approach does not guarantee that all electronic properties, e.g. *d* band widths or defect levels, are correct [9]. Recently, it has been argued from the screening behaviour of nonmetallic systems that *α* can be related to the inverse of the static dielectric constant [10, 11]

3 $$  \alpha = \frac{1}{\epsilon_{\infty}},  $$

which may then be computed in a self-consistent cycle [5, 12]. This iteration to self-consistency requires additional computational effort, but removes the empiricism of previous hybrid functionals and restores the ab initio character of the calculations. The utility of this approach, however, depends on the accuracy of the resulting predicted material properties. Here, we are interested in the implications for the structural, electronic, and optical properties of oxide semiconductors, and consider ZnO, SnO_2_, and MgO as an illustrative set of materials.

### Computational Details

The calculations presented in this work have been performed using the projector-augmented wave (PAW) method [13], as implemented in the Vienna ab initio simulation package (VASP 5.4.1) [14–16]. For the calculation of structural and electronic properties, standard PAW potentials supplied with VASP were used, with 12 valence electrons for Zn atom (4*s* ^2^3*d* ^10^), 14 valence electrons for Sn (5*s* ^2^4*d* ^10^5*p* ^2^), 8 valence electrons for Mg (2*p* ^6^3*s* ^2^), and 6 valence electrons for O (2*s* ^2^2*p* ^4^), respectively. When calculating dielectric functions, we have used the corresponding *GW* potentials, which give a better description of high-energy unoccupied states.

To evaluate the performance of the self-consistent hybrid approach, we have calculated structural and electronic data using three functionals: GGA in the PBE parametrisation [7], the hybrid functional PBE0 [8], and the self-consistent hybrid functional [5], which we denote scPBE0.

Structural relaxations were performed for the regular unit cells within a scalar-relativistic approximation, using dense *k* point meshes for Brillouin zone integration (8×8×6 for wurtzite ZnO, 6×6×8 for rutile SnO_2_, and 10 × 10 × 10 for rocksalt MgO). For each material, we performed several fixed-volume calculations, in the cases of ZnO and SnO_2_ allowing internal structural parameters to relax until all forces on ions were smaller than 0.001 eV Å^−1^. Zero-pressure geometries were determined by then fitting a cubic spline to the total energies with respect to the unit cell volumes.

To evalutate the self-consistent fraction of Hartree-Fock exact-exchange, *α*, the dielectric function *ε*_*∞*_ is calculated in an iterative series of full geometry optimisations. To calculate *ε*_*∞*_, for each of the ground state structures, the static dielectric tensor has been calculated (including local field effects) from the response to finite electric fields. For non-cubic systems (ZnO, SnO_2_), *ε*_*∞*_ was obtained by averaging over the spur of the static dielectric tensor $\frac {1}{3}\left (2\epsilon _{\infty }^{\perp }+\epsilon _{\infty }^{\parallel }\right)$. We have considered *ε*_*∞*_ to be converged when the difference between two subsequent calculations falls below ±0.01 [5].

## Results and Discussion

### Structural properties

**Fig. 1:**
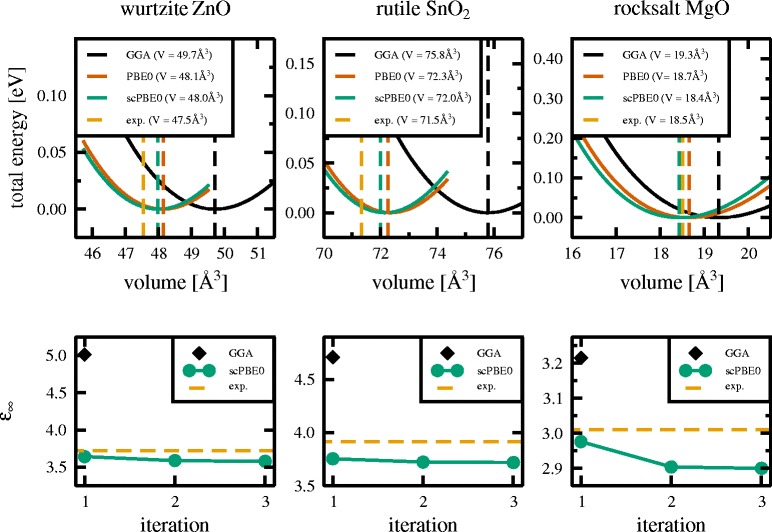
*Upper panels*: total energy (in eV) with respect to the unit cell volume for wurtzite ZnO (*left panel*), rutile SnO_2_ (*middle panel*), and rocksalt MgO (*right panel*) calculated by means of GGA (*black*), PBE0 (*red*), and scPBE0 functionals (*green*), respectively. The experimental unit cell volume is depicted by the *dashed orange line*. *Lower panels*: convergence for the dielectric constant *ε*_*∞*_ is obtained after three steps in the additional self-consistency cycle

ZnO crystallises in the hexagonal wurtzite structure of space group P6_3_mc (No. 186). SnO_2_ crystallises in the tetragonal rutile structure of space group P4_2_/mnm (No. 136). MgO crystallises in the cubic rocksalt structure of space group Fm$\bar {3}$m (No. 225). Each crystal structure was first fully geometry optimised, as described in the computational details section. The energy/volume data for the GGA, PBE0, and scPBE0 exchange-correlation potentials are plotted in the upper panels of Fig. 1. The GGA functional significantly overestimates the ground state volume relative to experimental values for all three materials. This is due to shortcomings in this simpler early exchange-correlation potential. The PBE0 functional adds a fixed proportion of Hartree-Fock exact-exchange (*α*=0.25) and produces structural properties in much better agreement with experimental data.

For our scPBE0 calculations, for each material, the static dielectric constant converged in three iterations (Fig. 1, lower panels). Here, computationally the most expensive part is the full geometry optimisation using the PBE0 functional. Each subsequent step in the self-consistent loop to determine the amount of Hartree-Fock exact-exchange starts from optimised crystal structures of the previous step and reduces the computational costs considerably.

Using the self-consistent amount of Hartree-Fock exact-exchange in the self-consistent hybrid functional yielded structural properties in slightly better agreement with experimental data (Fig. 1, upper panels). The improved description of structural properties using the scPBE0 functional is also evident from the lattice constants *a* (and *c*), which are given together with those obtained with the other two functionals and experimental data in Table 1.

**Table 1:** Ground state structural parameters for wurtzite ZnO, rutile SnO_2_, and rocksalt MgO obtained with different approximations for the exchange-correlation potential in comparison to low-temperature experimental data

ZnO	GGA	PBE0	scPBE0	Exp.
*a* [Å]	3.289	3.258	3.255	3.248 [20]
*c* [Å]	5.308	5.236	5.230	5.204 [20]
*u*	0.381	0.381	0.381	0.382 [21]
*ε*_*∞*_	5.01	3.64	3.58	3.72 [22]
*α*	−	0.25	0.28	−
*E*_gap_ [eV]	0.715	3.132	3.425	3.4449 [17]
SnO_2_	GGA	PBE0	scPBE0	Exp.
*a* [Å]	4.834	4.757	4.752	4.737 [23]
*c* [Å]	3.244	3.193	3.190	3.186 [23]
*u*	0.307	0.306	0.306	0.307 [23]
*ε*_*∞*_	4.71	3.76	3.72	3.92 [24]
*α*	−	0.25	0.27	−
*E*_gap_ [eV]	0.609	3.591	3.827	3.596 [18]
MgO	GGA	PBE0	scPBE0	Exp.
*a* [Å]	4.260	4.211	4.193	4.199 [25]
*ε*_*∞*_	3.22	2.98	2.90	3.01 [26]
*α*	−	0.25	0.34	−
*E*_gap_ [eV]	4.408	7.220	8.322	7.833 [19]

**Fig. 2:**
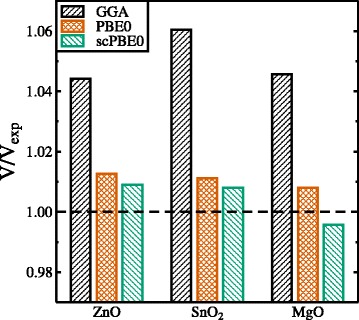
Ground state unit cell volumes *V* with respect to the experimental volume *V*_exp_ calculated by means of the GGA (*black*), PBE0 (*red*), and scPBE0 functionals (*green*), respectively. The experimental volumes correspond to *V*/*V*_exp_=1 (*dashed horizontal line*)

The quality of the structural data compared to experiment can also be seen from Fig. 2 where the coefficients of the different obtained ground state volumes with respect to the experimental one are plotted for the three different oxides. Again, the results obtained with the new self-consistent hybrid functional show closest agreement with experiment.

### Electronic and Optical Properties

**Fig. 3:**
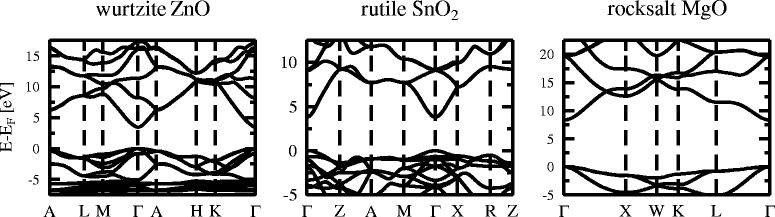
Electronic band structures of wurtzite ZnO (*left panel*), rutile SnO_2_ (*middle panel*), and rocksalt MgO (*right panel*), calculated with the scPBE0 functional. Energies are in electron volt (eV) and the valence band maximum is set to zero

**Fig. 4:**
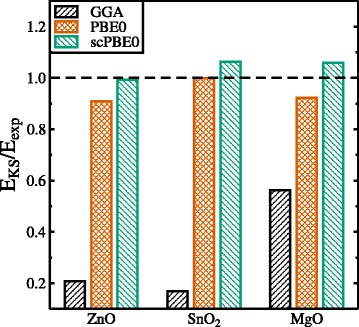
Ground state Kohn-Sham band gaps *E*_KS_ with respect to the experimental band gap *E*_exp_ calculated by means of the GGA (*black*), PBE0 (*red*), and scPBE0 functionals (*green*), respectively. The experimental band gaps correspond to *E*_KS_/*E*_exp_=1 (*dashed horizontal line*)

Figure 3 shows electronic band structures calculated using scPBE0 for wurtzite ZnO, rutile SnO_2_, and rocksalt MgO. The calculated (versus experimental) direct band gaps are 3.425 eV (3.4449 eV [17]) for ZnO, 3.827 eV (3.596 eV [18]) for SnO_2_, and 8.322 eV (7.833 eV [19]) for MgO, respectively (Table 1). Figure 4 shows the GGA, PBE0, and scPBE0 calculated band gaps alongside the experimental values. It can be seen that PBE0 calculated band gaps are underestimated compared to the experimental ones for all three oxides, but being very close for SnO_2_. The scPBE0 calculated band gaps are larger than the PBE0 values, thereby improving the results for ZnO and MgO, but worsen the result for SnO_2_. In general, the band gaps calculated using the hybrid functionals (PBE0, scPBE0) are within ten per cent of the experimental band gaps.

The scPBE0 calculations provide accurate structural properties and band gaps versus experimental data, and we can therefore be relatively confident when calculating properties less easily accessible directly by experiment. We have calculated the real (*ε*_1_) and imaginary (*ε*_2_) parts of the dielectric functions via Fermi’s Golden rule summing over transition matrix elements. For these calculations, we used the recommended VASP *GW* pseudopotentials and considerably increased the number of empty bands to ensure converged results.

**Fig. 5:**
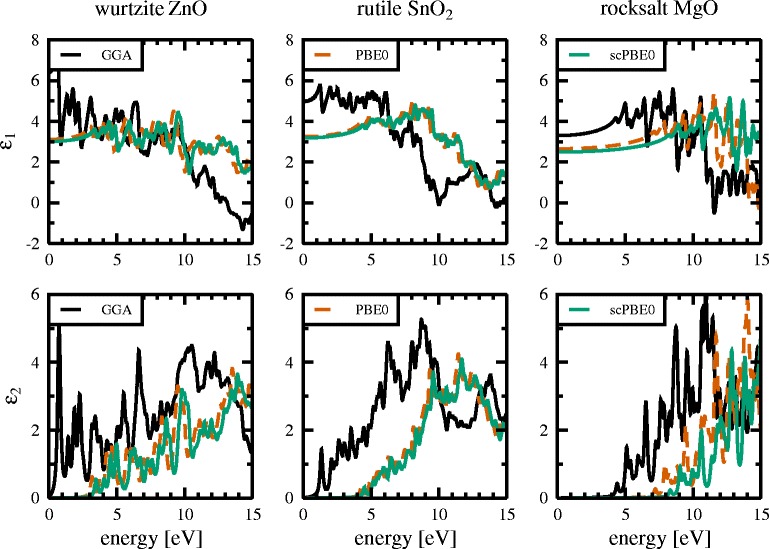
Real *ε*_1_ (*upper panels*) and imaginary *ε*_2_ (*lower panels*) parts of the dielectric functions calculated by means of GGA (*black*), PBE0 (*dashed red*), and scPBE0 functionals (*green*), respectively. Dielectric functions are shown for wurtzite ZnO (*left panels*), rutile SnO_2_ (*middle panels*), and rocksalt MgO (*right panels*)

Figure 5 shows the real (*ε*_1_) and imaginary (*ε*_2_) parts of the dielectric functions calculated with the GGA, PBE0, and scPBE0 functionals. Because the GGA functional significantly underestimates the band gap, the imaginary parts of the dielectric functions exhibit an onset, corresponding to the first allowed direct transition at the fundamental band gap, at lower energies. The onset energy improves considerably when switching to the PBE0 hybrid functional and improves further compared to experiment when using the self-consistent hybrid functional. For the two hybrid functionals, the overall shape of the real and imaginary parts of the dielectric functions are very similar in their peak structure but differ compared to the pure GGA functional. One reason for this difference might be the improvements in the *d* band width and position when using the hybrid functionals compared to the pure GGA one. Clarifying this would require a more in-depth comparison of the different band structures and how their specific features influence the dielectric functions.

## Conclusions

We have presented a theoretical investigation on the application of a new self-consistent hybrid functional to oxide semiconductors ZnO, SnO_2_, and MgO. We have presented and compared calculated structural, electronic, and optical properties of these oxides to experimental data, and have discussed the implications of using the new self-consistent hybrid functional. We find that the self-consistent hybrid functional gives calculated properties with accuracies as good as or better than the PBE0 hybrid functional. The additional computational cost due to the self-consistency cycle is justified by avoiding the empiricism of similar hybrid functionals, which restores the ab initio character of these calculations.

## Abbreviations

DFT: Density functional theory
GGA: Generalised gradient approximation
PAW: Projector-augmented wave
PBE: Perdew, Burke, and Ernzerhof
TCOs: Transparent conducting oxides
VASP: Vienna ab initio simulation package
